# Effectiveness of topical application with dexamethasone during sagittal split osteotomy of the mandible in minimising clinical symptoms of postoperative neurosensory disorders

**DOI:** 10.1186/s12893-025-02803-1

**Published:** 2025-02-20

**Authors:** Iryna Logvynenko, Larysa Dakhno, Valeriia Bursova

**Affiliations:** 1 Department of Oral and Maxillo-Facial Surgery, Bogomolets National Medical University, Kyiv, 01601 Ukraine; 2 Central Laboratory Diagnosis of the Head, Kyiv, 04080 Ukraine

**Keywords:** Orthognathic surgical procedures, Mandibular nerve injuries, Sensation disorders, Sensitivity, Inferior alveolar nerve

## Abstract

**Background:**

The aim of this study was to investigate the effectiveness of topical application with dexamethasone during BSSO due to its anti-inflammatory effect and decreasing of postoperative nerve oedema in minimising clinical symptoms of NSD associated with inferior alveolar nerve (IAN) injury in postoperative period, based on the results of sensory diagnostic tests, such as light touch test and its modifications.

**Methods:**

Through randomisation, 2 groups were selected from 22 patients: the experimental– where topical application with solution of dexamethasone phosphate 0.4% (4 mg/1 ml ampules) during sagittal split osteotomy of the mandible was used, and the control– where the classical technique of BSSO was held.

**Results:**

Authors performed diagnostics of NSD using Light Touch test on 1st day, 1st week, 1st month, 3rd month and 6th month postoperatively. The experimental group showed improvements in sensory recovery compared to the control group, particularly from 1 week to 3 months post-surgery. By 6 months, both groups achieved similar levels of sensitivity restoration.

**Conclusions:**

This confirms the effectiveness of proposed method and opens up further prospects for the updated function of the IAN following the BSSO.

**Clinical trial number:**

Not applicable.

## Introduction

Bilateral sagittal split osteotomy (BSSO) is a standard procedure in orthognatic surgery for correcting skeletal deformities of the mandible [1, 2]. Despite the existence of a wide variety of modifications to this surgical technique, researchers report in their studies that the frequency of postoperative NSD in the areas of the lower lip and chin ranges from 9 to 85% [3, 4]. In many cases, NSD completely disappear 6 months after the BSSO, but sometimes the symptoms of sensory loss can persist for up to a year or even stay permanent, impacting the patient’s well-being and reducing their quality of life [5, 6]. The choice of 6 months as the endpoint in this study was based on the typical recovery trajectory for NSD following BSSO. It is common to observe significant improvement in NSD within the first six months post-surgery, and this timeframe is often used to evaluate the effectiveness of interventions aimed at accelerating recovery, such as the application of dexamethasone.

However, it is acknowledged that some patients may continue to experience improvements in neurosensory function beyond six months, with full recovery sometimes occurring between 6 months and 1 year after surgery. The authors chose a 6-month endpoint because it represents a reasonable window during which the majority of NSD improvement occurs, making it an appropriate time for evaluating the initial effects of the treatment. Additionally, a six-month follow-up period allows for an assessment of both the immediate benefits of the intervention and any potential complications or residual effects.

Evaluation of NSD symptoms resulting from inferior alveolar nerve (IAN) injury after BSSO of mandible, which includes the chin and lower lip areas, can be conducted through different methods: subjective (questionnaires, where the patient should answer the several questions to determine whether they have any sensory loss or not), relatively objective (such as a brush directional stroke test, 2-point discrimination test, thermotesting, static light touch test (LT)), and purely objective (for instance, a blink reflex test, trigeminal somatosensory evoked potentials, sensory nerve action potential) [7, 8]. Furthermore, L.J. Poort et al. [9] reports, that over 50% of the BSSO studies described LT and TPD tests as basic tools in diagnosing NSD.

To date, there is no unequivocal opinion regarding the optimal treatment tactics for patients with possible NSD associated with IAN injury following BSSO [10]. P. Coulthard et al. [11] outlined various interventions for treating peripheral nerve damages, including conservative therapy, surgical treatment methods (neurorrhaphy, neurolysis (internal/ external), excision of neuromas, nerve autografts, and tubulisation), common counselling approaches (psychotherapy and sensory re-education exercises), and low-level laser therapy. E.B. Galloway et al. [12] in their study, investigated the effectiveness of local use of dexamethasone in minimising NSD following peripheral nerve injury of the axonotmesis type (2d degree of severity according to Seddon’s classification). They found that using dexamethasone in the form of application significantly enhanced nerve recovery 14 days after damage in rats. Furthermore, H. Suslu et al. [13] conducted research involving 32 sciatic nerves injuries of rats and concluded that topical application of dexamethasone was more effective than its systemic administration. However, F. Pourdanesh et al. [14] reported contrasting findings in their study, where they noted that local use of dexamethasone on the exposed area of IAN during a BSSO of the mandible did not lead to therapeutic effect in restoration of nerve function. They suggested that the lack of efficacy might be attributed to the method of usage– multiple irrigations performed during surgery, potentially leading to the removal of dexamethasone and insufficient absorption time.

Finally, the effectiveness of local therapy with dexamethasone on IAN regeneration has not been studied enough due to the deficiency of sufficient human clinical trials. This highlights the relevance of further research in this field. To investigate the effectiveness of topical application with dexamethasone during BSSO in minimising clinical symptoms of NSD associated with IAN injury in postoperative period, based on the results of sensory diagnostic tests.

## Materials and methods

In order to participate in the study, all participants had to sign an informed consent. Patients of both groups received systemic dexamethasone in postoperative period according to the following regimen: 12 mg at 1 day after surgery, 8 mg at 2 day, 4 mg at 3 and 4.5 days after surgery. Inclusion criteria: patients over 18 years old with a diagnosis of skeletal malocclusion that will require bilateral sagittal split osteotomy (BSSO) surgery, patients with normal sensitivity of lower lip and chin areas in preoperative period, patients with IAN exposure on the both sides during BSSO. A traditional surgical technique was used to perform a BSSO. The operation was performed using standard surgical instruments for osteotomy, including scalpels, saws and specialized instruments for correcting the position of the bones. This method allows to achieve an accurate and stable result, minimizing the risk of complications. The choice of this technique was justified by the optimal balance between efficiency and minimization of injuries to surrounding tissues. A computer-generated list of random numbers was used to randomize participants. This method ensures an unbiased distribution of participants between the control and experimental groups. Using a computer algorithm, each patient was assigned a random number that determined their assignment to one of the groups. This approach ensures that no external subjectivity affects the allocation of participants, which increases the reliability and validity of the results.

To perform the osteotomy, the method of division was used with the help of surgical dilators and special osteotomy instruments, which allows to create the necessary bone dissection. This method has its advantages in that it reduces the risk of damage to nerve structures and ensures accurate and controlled separation of bone fragments. In addition, surgical instruments allow the operation to be performed with minimal trauma to the surrounding tissues, which is important for faster healing and reduced postoperative complications. During the operation, no partial or complete resection of the inferior alveolar nerve was observed. The nerve structures were carefully evaluated during surgery to detect possible damage or compression. To prevent damage to the nerve, methods of careful manipulation of bone fragments and careful monitoring of the nerve structures were used. The use of appropriate tools and techniques reduces the likelihood of nerve injury, which is critical for maintaining normal sensation in the postoperative period.

Before closing the wound, a thorough irrigation of the surgical field was performed using saline solution to cleanse it of residual tissue, blood and other contaminants. This procedure helps reduce the risk of infection and improves the healing process. Irrigation also allows you to check for tissue damage and provide the best conditions for rapid healing. No drainage was placed after the operation, as all patients demonstrated satisfactory healing results without the need for additional drainage. Postoperative follow-up showed that there was no need to remove fluid from the surgical site, as there were no complications such as fluid accumulation or hematomas. Patients had satisfactory healing without the need for additional interventions.

**Fig. 1:**
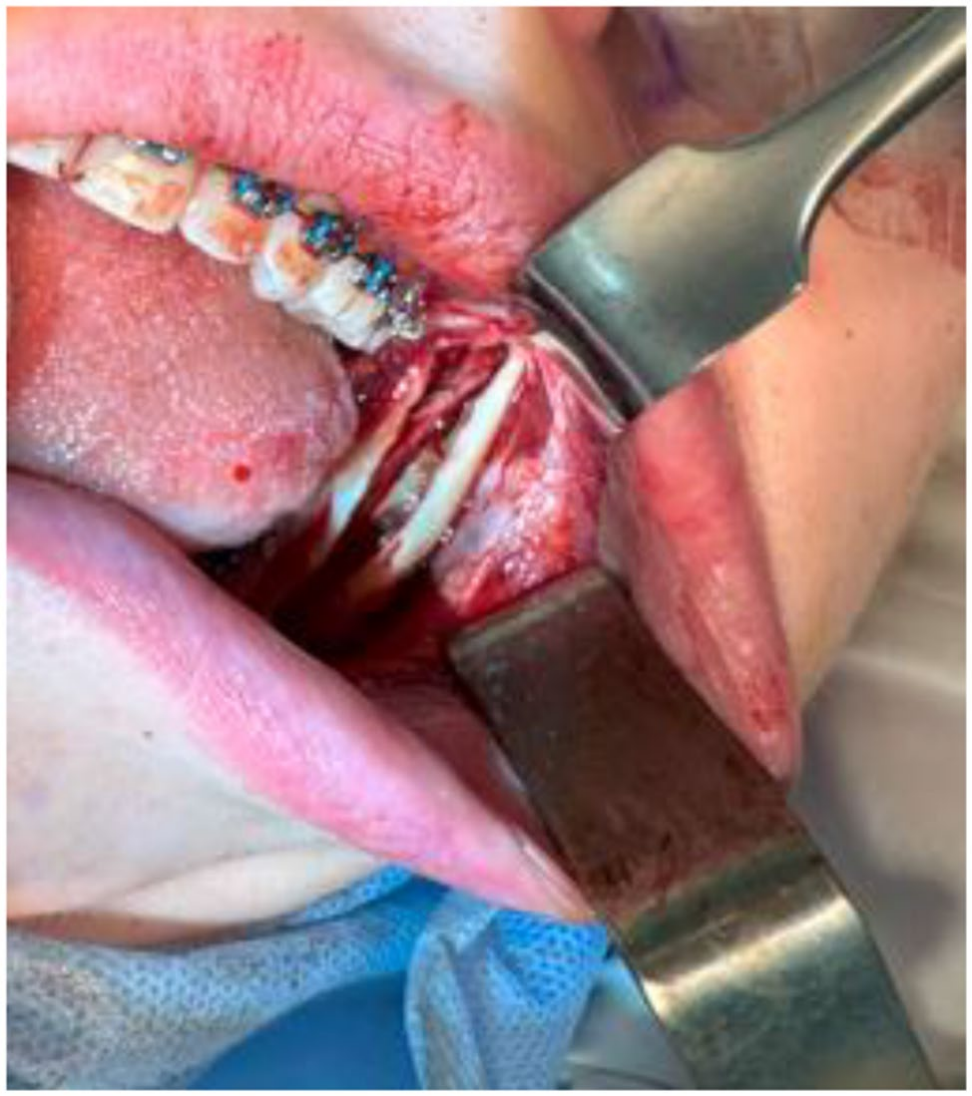
Area of the exposed left IAN. *Source* compiled by the authors

**Fig. 2:**
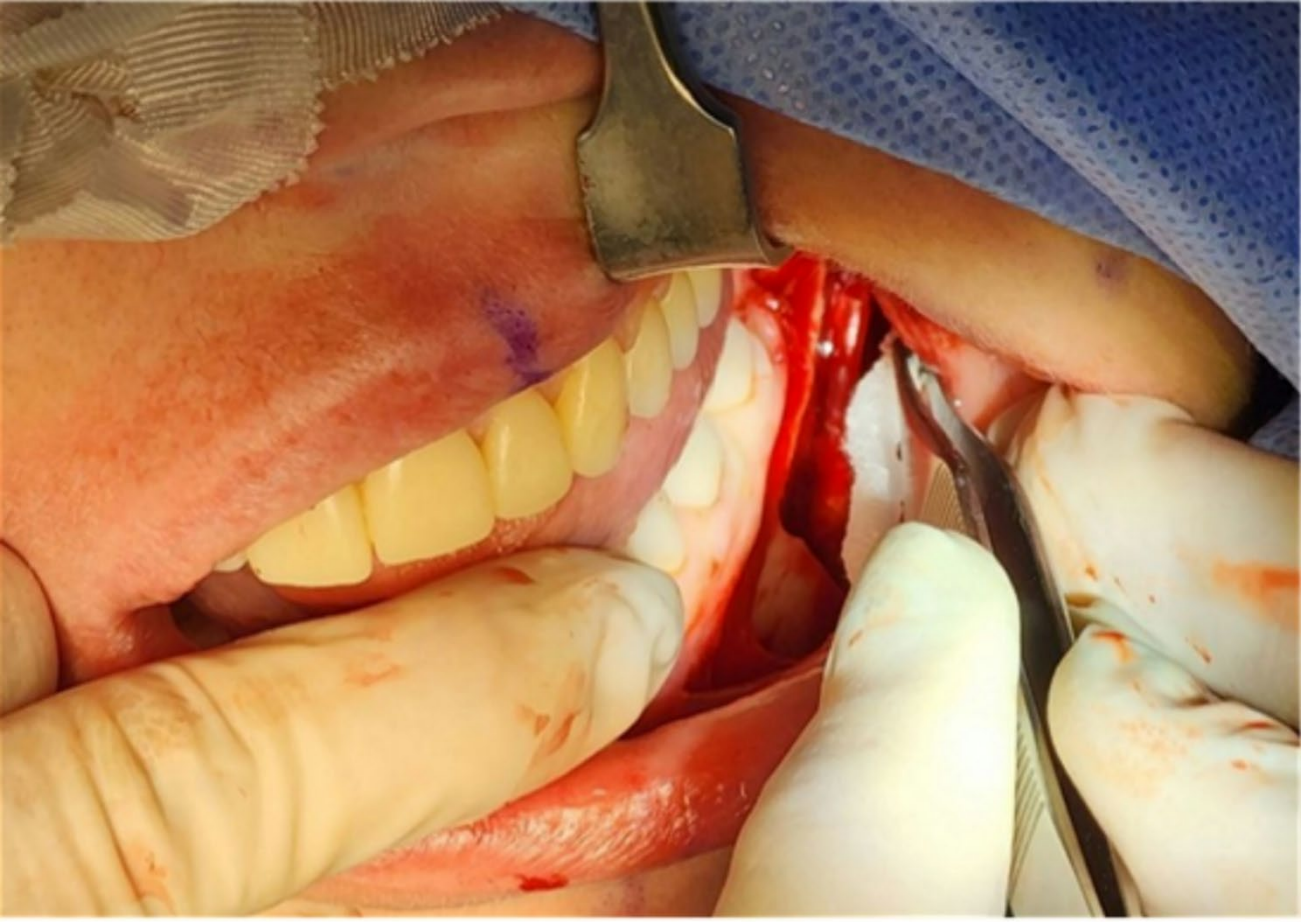
Method of application, using gauze turunda. *Source* compiled by the authors

Exclusion criteria: patients with previous surgical interventions in the mandible, patients with injury of mandible in anamnesis, patients with new lesions in the mandible, patients with mental disorders, patients, who underwent BSSO without any involvement of the IAN. In this study, authors suggested a protocol for the local application of dexamethasone phosphate solution. One team of maxillofacial surgeons performed 44 BSSO on 22 patients using the same method. The control group (*n* = 11) and experimental group (*n* = 11) were randomly selected. A solution of dexamethasone phosphate 0.4% (4 mg/1 ml ampules) was used. A gauze turunda soaked with the solution was applied to the area of the exposed IAN after BSSO and mobilisation of bone fragments for 10 min before osteosynthesis (Figs. 1 and 2). The occurrence of NSD and its evaluation was tested after orthognathic surgery.

**Fig. 3:**
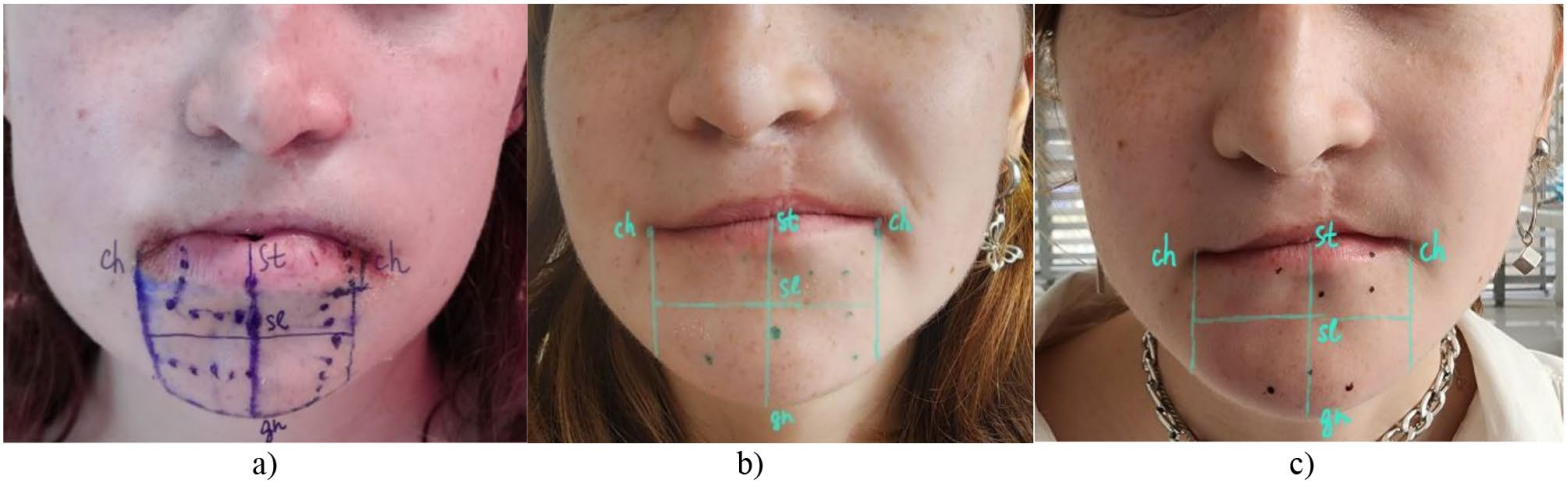
Marking of diagnostic zones: **a**) 1 day after surgery; **b**) 1 week after surgery; **c**) 1 month after surgery. *Source* compiled by the authors

We performed diagnostics of NSD using LT test [9] at 1st day, 1st week, 1st month, 3rd month, and 6th month postoperatively. Firstly, 2 zones were marked with a solid line: on the lower lip– from landmark Stomion (st) to Mentolabial sulcus (ms) (innervation– labial branch of IAN), on the chin– from landmark ms to Gnation (gn) (innervation– mental branch of IAN) and, then divided into 4 segments– right and left of each zone, focusing on the cental line of the face and vertical lines from landmarks Chelion (ch) (Fig. 3).

**Fig. 4:**
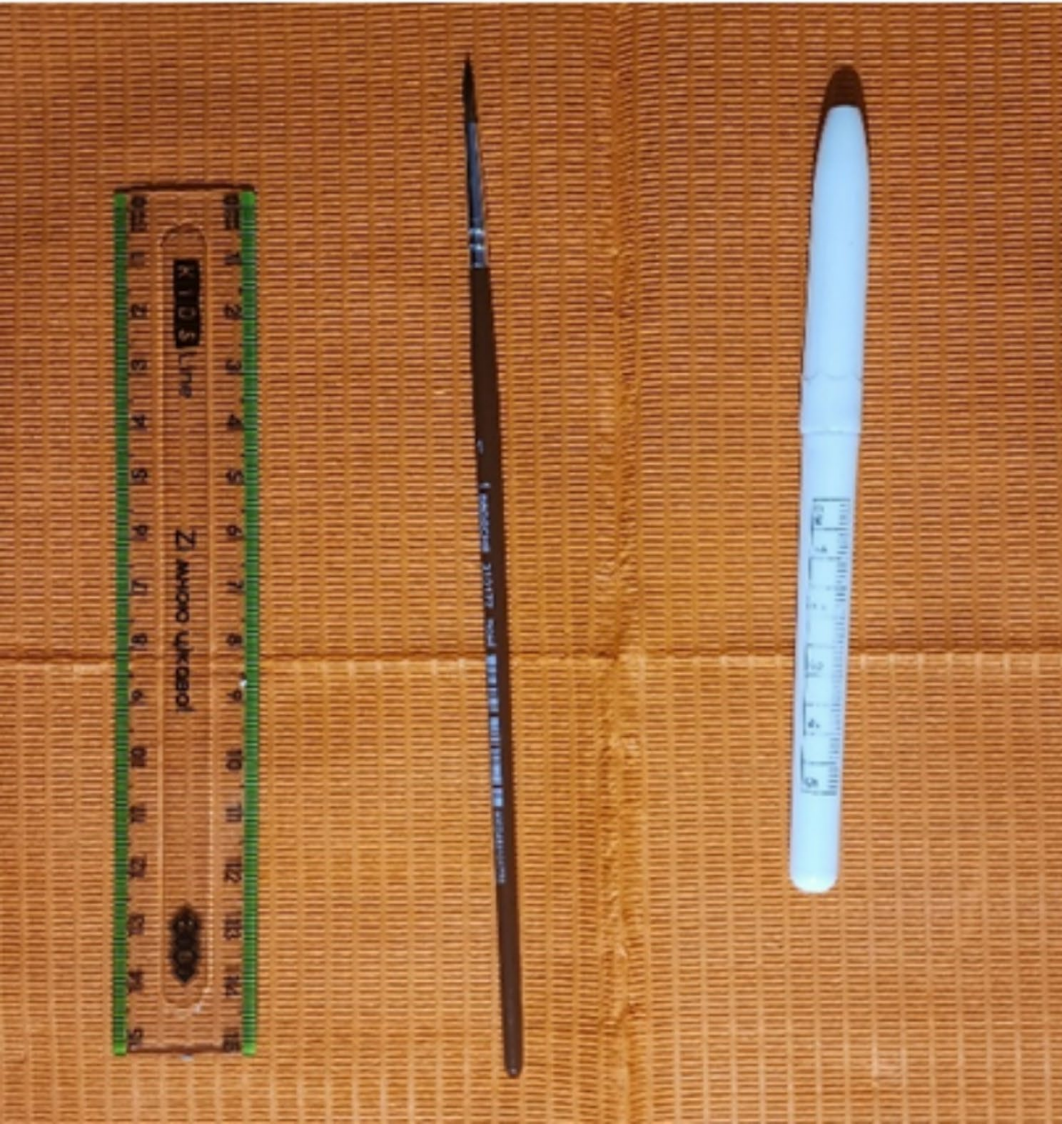
Tools for measurements. *Source* compiled by the authors

The assessment of each segment was conducted thrice, with a true response being defined as consistent outcomes in at least two out of three examinations, according to the protocol outlined by P.G. Antony et al. [7]. During this diagnostic procedure, patients were requested to close their eyes to determine more precisely boundaries of paraesthesia areas. Tools that were used are shown in the Fig. 4.

The examined person is in a sitting position with closed eyes. Using a tassel, a touch is applied to each of the 4 segments, repeated 3 times. The patient communicates with the examiner by raising a hand in response to the stimulus. Test outcomes are compared with those obtained by touching an undamaged area (in this case, the cheek area was selected). We also used the tassel to touch the healthy area (cheek area) and move it to the lower lip and chin zones at different levels. The patient signalled when they stopped feeling the touch by raising their hand. Authors marked the boundaries of paraesthesia with a dotted line and recorded distance. Statistical processing of data was performed in the EZR, MedStat software using Shapiro-Wilk test, non-parametric Mann-Whitney U test for independent samples, non-parametric Friedman and Wilcoxon tests for related samples. A *p* < 0.05 was considered statistically significant.

## Results

In the present study, genioplasty was not performed simultaneously with BSSO surgery. The osteotomy surgery was the main one, and the changes in sensitivity were directly related to BSSO. All patients did not receive additional surgical interventions that could lead to an increased risk of neurosensory disorders (NSD). No postoperative complications such as infection or improper bone fusion were observed. All patients demonstrated satisfactory healing without signs of infection. The bone fusion process proceeded without any abnormalities, and based on careful postoperative follow-up and control examinations, no abnormalities such as hematomas or other problems that could affect the outcome of treatment were detected.

**Fig. 5:**
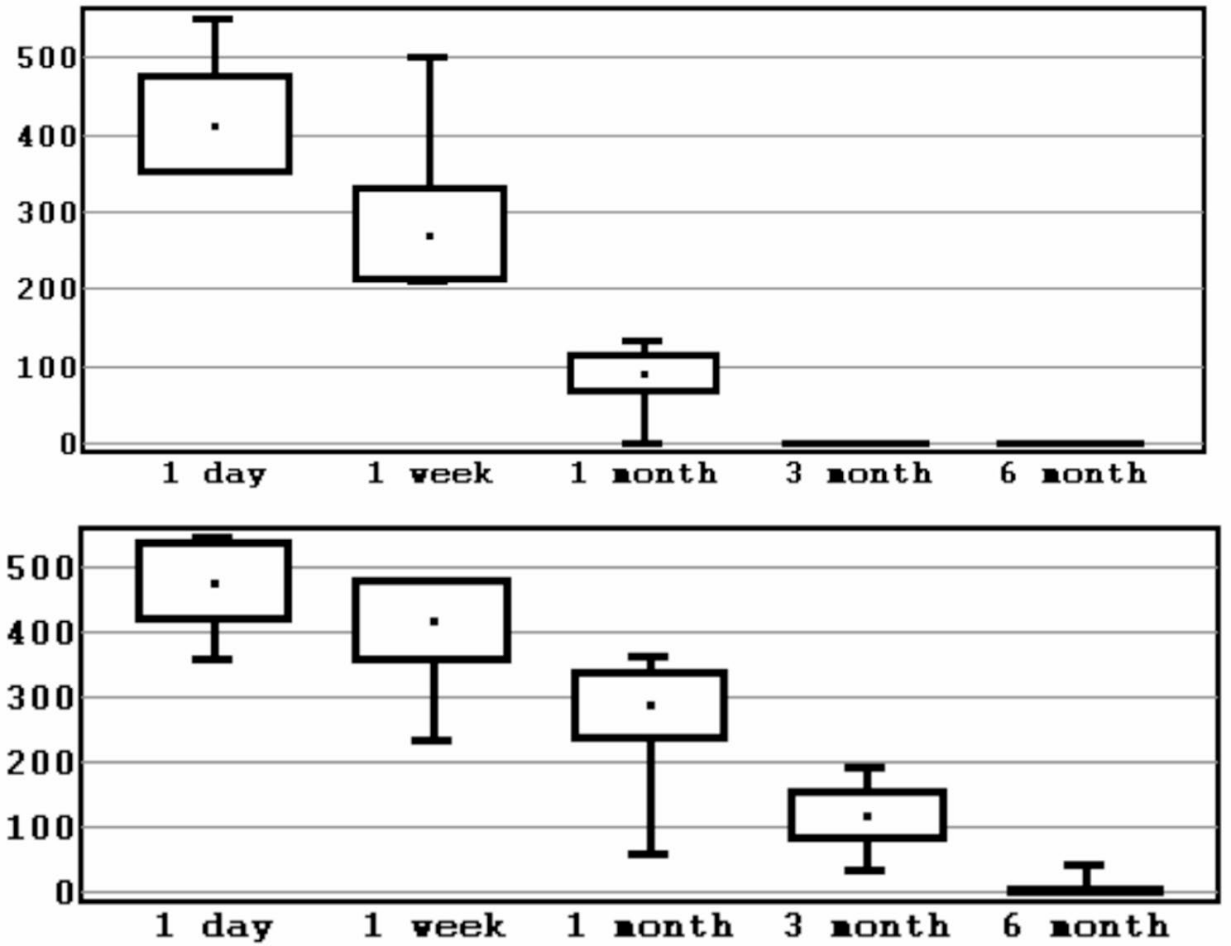
Interval estimation of the mean values of sensitivity loss indicator (mm^2^). *Note* mean values, error of the mean, 95% confidence interval (experimental/control group,1 segment). *Source* compiled by the authors

**Fig. 6:**
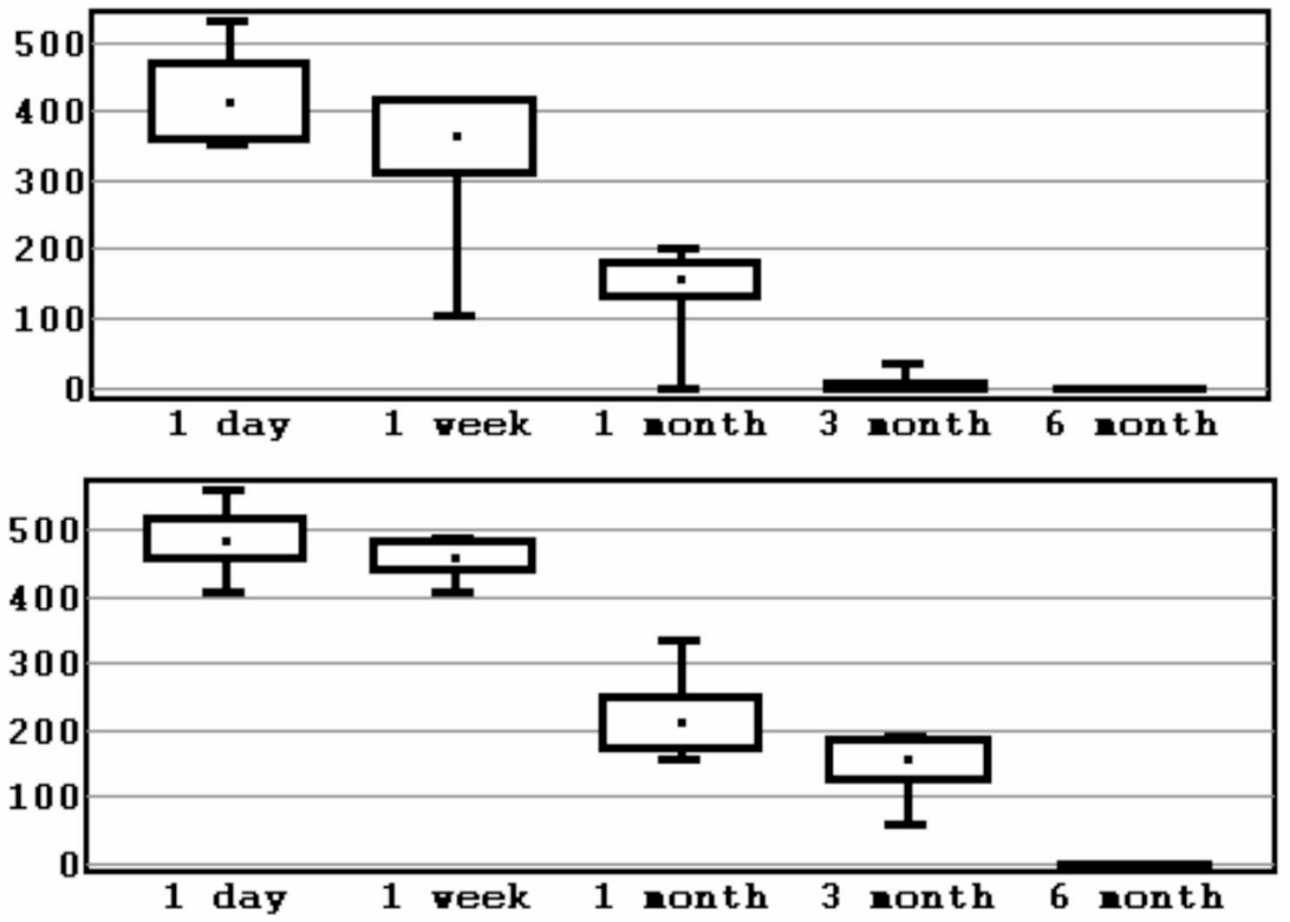
Interval estimation of the mean values of sensitivity loss indicator (mm^2^). *Note* mean values, error of the mean, 95% confidence interval (experimental/control group, 2 segment). *Source* compiled by the authors

**Fig. 7:**
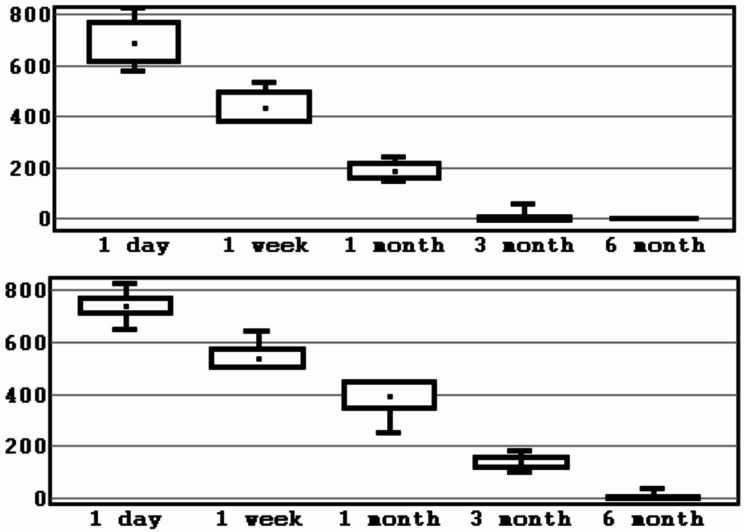
Interval estimation of the mean values of sensitivity loss indicator (mm^2^). *Note* mean values, error of the mean, 95% confidence interval (experimental/control group, 3 segment). *Source* compiled by the authors

**Fig. 8:**
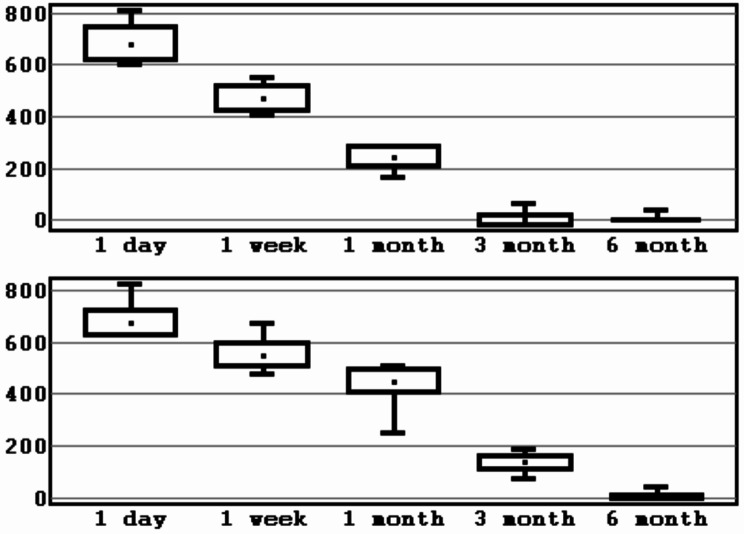
Interval estimation of the mean values of sensitivity loss indicator (mm^2^). *Note* mean values, error of the mean, 95% confidence interval (experimental/control group, 4 segment). *Source* compiled by the authors

The total number of patients was 22, of which 13 were males and 9– females. Mean age was 30.4 ± 7.8 years (Table 1). A total of 44 BSSO were performed. As a result of checking the distribution of indicators of loss of sensitivity using Shapiro-Wilk test, it was established that the distribution differs from the normal one at the significance level of *p* < 0.05. To present the data, the mean value and the standard deviation (M ± SD) were calculated for the experimental group and the control group (Table 2; Figs. 5, 6, 7 and 8).

**Table 1:** Distribution of patients by gender and incidence of NSD

Gender	*N* of patients	*N* of BSSO	% of NSD incidence
Male	13	26	59.09%
Female	9	18	40.91%

*Source* compiled by the authors

**Table 2:** Point estimation of the mean values of sensitivity loss indicator (mm^2^) of segments 1–4 in the experimental and control groups over 1 day, 1 week, 1 month, 3 months, and 6 months after BSSO

Sensory test terms	Group	*N* of BSSO	Mean value of sensitivity loss in 1 segment, M ± SD, mm^2^	Mean value of sensitivity loss in 2 segment, M ± SD, mm^2^	Mean value of sensitivity loss in 3 segment, M ± SD, mm^2^	Mean value of sensitivity loss in 4 segment, M ± SD, mm^2^
1 day	Experimental	22	416.6 ± 167.5	393.2 ± 217.7	653.8 ± 244.3	637.4 ± 234.1
	Control	22	425.6 ± 162.4	476.6 ± 84.5	737.5 ± 107.9	662.3 ± 242.9
7 days	Experimental	22	307.5 ± 161.4	320 ± 207.9	432 ± 178.6	438.3 ± 175.5
	Control	22	333.5 ± 168.7	440.3 ± 62.5	576.5 ± 125.3	550 ± 225.1
1 month	Experimental	22	72.5 ± 64.7	138.8 ± 108.7	178.6 ± 98.1	215.5 ± 120.3
	Control	22	227 ± 139.8	241.2 ± 103.4	385.5 ± 176.9	363.6 ± 195.8
3 months	Experimental	22	0	10.7 ± 26.2	14 ± 32.3	6.3 ± 20.8
	Control	22	115.7 ± 97.4	126.8 ± 80.6	130.6 ± 66.7	132.5 ± 109.9
6 months	Experimental	22	0	0	0	0
	Control	22	3.6 ± 12	0	9.6 ± 22.2	10.1 ± 33.5

*Source* compiled by the authors

**Fig. 9:**
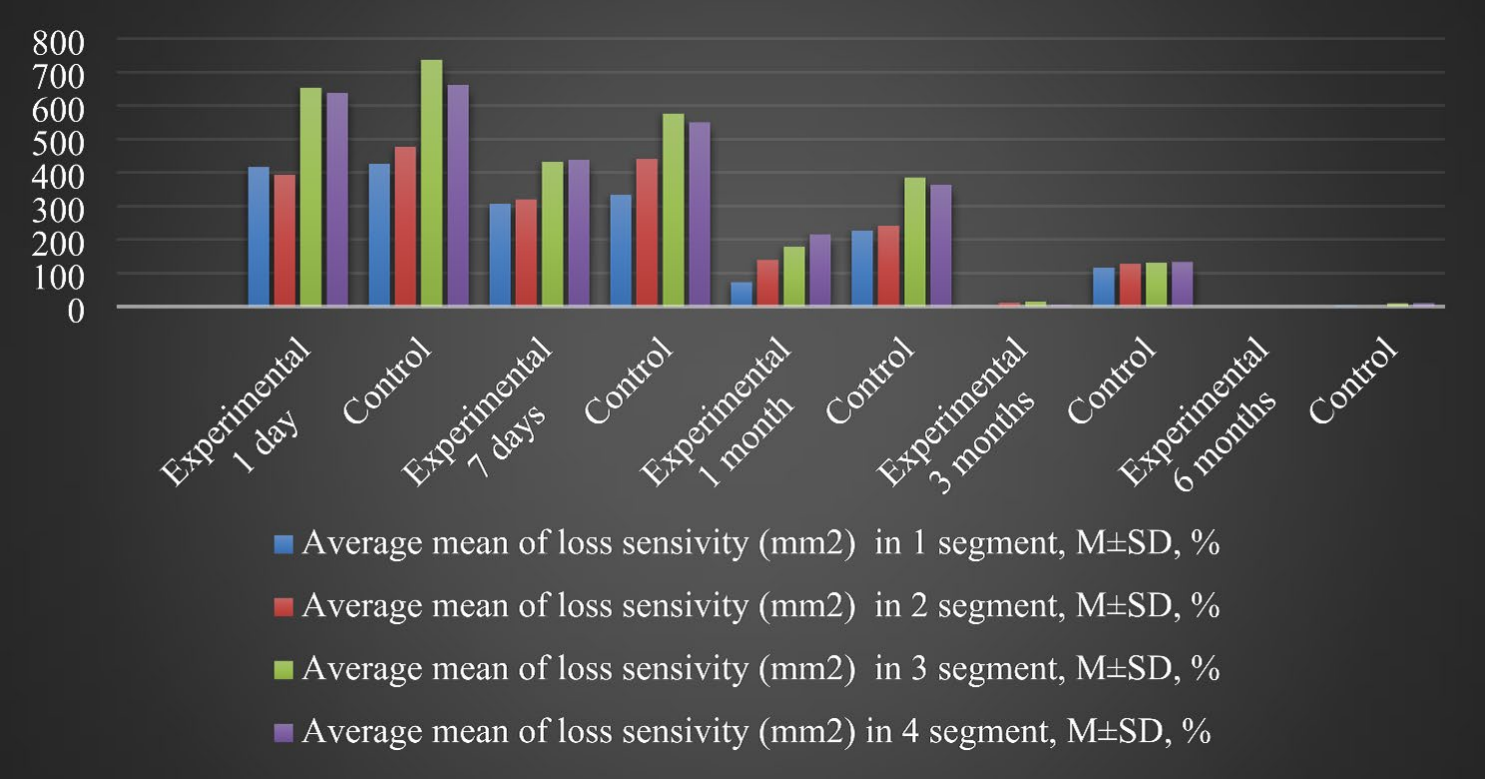
The dynamics of changes in sensitivity loss indicator among the experimental and control groups over 1 day, 1 week, 1 month, 3 months, and 6 months after BSSO. *Source* compiled by the authors

Next, the non-parametric Mann-Whitney U test was applied for independent samples. The p-values obtained for each sensory test term indicate the level of statistical significance in the differences between the two groups. At 1st day after BSSO difference in results of control and experimental groups was not statistically significant (*p* > 0.05). At 1 week after surgery area of neurosensory loss in 3 and 4 segments in experimental group was smaller compared to control group at *p* = 0.0281, *p* = 0.0939. Starting from 1 to 3rd month results in experimental group were significantly better, as evidenced by a statistically significant difference in 2 groups. By 6 months, both groups achieved similar levels of sensitivity recovery (Table 3; Fig. 9).

**Table 3:** The median values (me) of sensitivity loss indicator (mm^2^), the interquartile range (Q1-Q3), p values of 1–4 segments in experimental and control groups over 1 day, 1 week, 1 month, 3 months, and 6 months after BSSO

Sensory test terms	Segment	(Me, Q1-Q3), experimental group	(Me, Q1-Q3), control group	Experimental/Control group
1 day	1	356.5 (0-413)	384 (0-476)	*p* = 0.844
	2	413 (356.5-554.5)	485 (412–553)	*p* = 0.533
	3	688 (582–818)	806 (656.5–826)	*p* = 0.532
	4	688 (582–790)	672 (625–825)	*p* = 0.693
1 week	1	270 (210.5-443.5)	416 (279.5-451.5)	*p* = 0.669
	2	363 (194-461.5)	460 (412–481)	*p* = 0.148
	3	435 (371.5–523)	550 (512–650)	*p* = 0.0281
	4	454 (402.5–542)	611 (454-689.5)	*p* = 0.0939
1 month	1	91 (0-116)	286 (120–348)	*p* = 0.0132
	2	156 (42-206.5)	210 (175-328.5)	*p* = 0.0814
	3	188 (140.5–226)	416 (270-450.5)	*p* = 0.00186
	4	241 (134–259)	461 (251.5-511.5)	*p* = 0.0527
3 months	1	0 (0–0)	117 (48–164)	*p* = 0.000306
	2	0 (0–0)	155 (64-178.5)	*p* = 0.000642
	3	0 (0–0)	136 (95-180.5)	*p* = 0.000383
	4	0 (0–0)	131 (64-180.5)	*p* = 0.000751
6 months	1	0 (0–0)	0 (0–0)	*p* = 0.363
	2	0 (0–0)	0 (0–0)	p = NaN
	3	0 (0–0)	0 (0–0)	*p* = 0.167
	4	0 (0–0)	0 (0–0)	*p* = 0.363

*Source* compiled by the authors

As a result of the use of Friedman test for related samples with pairwise comparisons according to Bonferroni, the non-parametric Wilcoxon test for comparison, it was found that there was a statistically significant difference in results obtained between 1 and 2 segments at different postoperative intervals. At 1-month post-surgery in experimental group (*p* = 0.0423) and 1 week in control group (*p* = 0.03), neurosensory sensitivity in 1 segment was found over a larger area compared to segment 2. Conversely, no such significant differences were detected between segments 3 and 4 in either cohort (Table 4).

**Table 4:** P values of 1/2 and 3/4 segments in experimental and control groups over 1 day, 1 week, 1 month, 3 months, and 6 months after BSSO

Sensory test terms	Experimental group		Control group	
	1 segment/2 segment	3 segment/4 segment	1 segment/2 segment	3 segment/4 segment
1 day	*p* = 1	*p* = 1	*p* = 1	*p* = 1
1 week	*p* = 0.965	*p* = 0.359	*p* = 0.03	*p* = 0.646
1 month	*p* = 0.0423	*p* = 0.638	*p* = 0.831	*p* = 0.919
3 months	*p* = 0.371	*p* = 0.789	*p* = 0.966	*p* = 1
6 months	p = NaN	p = NaN	*p* = 1	*p* = 1

*Source* compiled by the authors

The use of dexamethasone in sagittal split osteotomy of the mandible proves to be highly effective in minimising postoperative neurosensory disorders. This drug has powerful anti-inflammatory properties, which helps to reduce swelling and reduce compression of nerve structures, such as the inferior alveolar nerve. This is especially important, as nerve compression after surgery is the main cause of temporary or permanent sensory disturbances, such as numbness or tingling sensations in the lips, chin, or teeth [8, 13, 14]. Studies have shown that the use of dexamethasone in topical application during sagittal split osteotomy of the mandible significantly reduces the period of recovery of inferior alveolar nerve function compared to patients who received only systemic dexamethasone. Patients, who undergoed topical application with dexamethasone during BSSO were more likely to demonstrate full recovery of sensation several months after surgery, while without the use of this drug intraoperatively, sensory disturbances could persist for up to six months. Dexamethasone is an effective tool for minimising clinical symptoms of postoperative neurosensory disorders and accelerating the recovery of nervous system functions in patients undergoing sagittal split osteotomy [15].

Dexamethasone acts as a powerful anti-inflammatory agent, reducing swelling in the surgical area, which puts pressure on nerve structures. Thus, the drug helps to minimise nerve compression and facilitates its faster recovery. This significantly reduces the duration of postoperative neurosensory disturbances, which can persist for a long time, up to several months without the use of dexamethasone [2]. By reducing inflammation and swelling, patients regain sensation faster and return to normal life. It also reduces the risk of chronic complications, which is especially important for ensuring a high quality of life for patients after surgery. In general, its use increases the effectiveness of treatment and reduces the likelihood of long-term or irreversible consequences for the nervous system. However, dexamethasone, like any powerful drug, has a number of side effects that arise due to its impact on various body systems. One of the main mechanisms of action of dexamethasone is the suppression of the immune system and anti-inflammatory effect, which leads to a violation of the body’s natural defence response [16, 17]. As a result, long-term use of dexamethasone may result in increased vulnerability to infections.

Other side effects include metabolic disorders. The drug can cause an increase in blood sugar levels, which is particularly risky for patients with diabetes. It may also cause fluid retention in the body, leading to oedema and high blood pressure. Long-term use can cause adrenal dysfunction, leading to Cushing’s syndrome, with characteristic signs such as obesity, moon-shaped face, thin skin, bruising, and muscle weakness. Other possible side effects include psycho-emotional disturbances, such as depression, insomnia or anxiety. Such reactions occur due to the impact on the central nervous system and hormonal balance [18–20]. Osteoporosis can also develop due to prolonged inhibition of bone synthesis. These side effects occur due to the systemic effect of dexamethasone on the body’s hormonal and metabolic balance, so it is important to use it in the lowest effective doses and under strict medical supervision.

If dexamethasone is not used for postoperative neurosensory disorders significant complications in the recovery of nerve function can occur. After a surgical procedure such as sagittal split osteotomy, swelling and inflammation are a natural response to the injury. Without the use of anti-inflammatory medications such as dexamethasone, the swelling can put pressure on the inferior alveolar nerve, resulting in prolonged or even permanent numbness, tingling and loss of sensation in the lip and chin area. Failure to properly control inflammation increases the risk of prolonged recovery of nerve function, which can take several months to a year or even longer. This not only significantly prolongs the period of discomfort for the patient, but also increases the likelihood of chronic complications, such as permanent loss of sensation or nerve damage [21, 22]. In addition, the lack of treatment can complicate the rehabilitation process, as patients may experience significant difficulties in everyday life due to sensory impairment. Thus, without dexamethasone, the risks of postoperative complications increase significantly, making its use an important component of successful treatment [23–25].

This type of research not only helps to improve the quality of life of patients, but is also important for the further development of surgical practice in the field of maxillofacial surgery. The results can form the basis for the introduction of new standards of local treatment during BSSO and will lead to a reduction in the number of complications and the duration of rehabilitation. In general, this study makes a significant contribution to the development of scientific knowledge about the prevention and management of postoperative neurosensory disorders, and opens up new opportunities for the effective treatment of patients with such pathologies. The use of this drug can significantly reduce postoperative pain and swelling, and improve functional outcome [26, 27]. The analysis of the data obtained indicates a positive effect of dexamethasone on the tissue healing process, but it is important to consider potential side effects. Further study of the optimal doses and regimens of dexamethasone administration is recommended to maximise its benefits in surgical practice. Given the results, dexamethasone may be a useful in a comprehensive approach to the management of postoperative symptoms in patients undergoing osteotomy.

## Discussion

The study of the effectiveness of topical dexamethasone in BSSO is extremely relevant due to the high incidence of postoperative neurosensory disorders that significantly affect the quality of life of patients. After BSSO, alveolar nerve damage is one of the most common complications, leading to temporary or long-term loss of sensation in the chin, lips, and gums. These disorders have a significant clinical impact, which can include discomfort, impaired chewing function and aesthetic inconvenience for patients.

Dexamethasone is a synthetic glucocorticoid with potent anti-inflammatory and immunosuppressive properties [28] Its action is to suppress the body’s immune response and reduce inflammation by inhibiting the production of prostaglandins, cytokines, and other inflammatory mediators. This is achieved by reducing the activity of phospholipase A2, which is responsible for the synthesis of arachidonic acid, a precursor of prostaglandins and leukotrienes involved in the development of inflammation.

Dexamethasone also affects the migration of leukocytes to the site of inflammation, reducing capillary permeability and stabilising cell membranes. Due to this, it reduces swelling, pain, and other signs of an inflammatory reaction. When used in maxillofacial surgery, dexamethasone effectively reduces postoperative swelling and accelerates tissue healing, which is important in complex surgeries such as mandibular osteotomy [29]. In addition to its anti-inflammatory effect, dexamethasone also reduces the risk of postoperative complications, such as numbness, due to its ability to reduce pressure on nerve structures by swelling reduction. This action makes it an indispensable tool in the treatment of patients during and after surgical interventions that involve work with bone structures, nerves, or soft tissues. Dexamethasone not only improves treatment outcomes and promotes faster recovery, but also reduces patient discomfort, helping to prevent long-term complications after surgery [30]. This study was conducted to determine its topical use effectiveness in sagittal split osteotomy.

Dexamethasone, as a potent glucocorticoid drug with pronounced anti-inflammatory and anti-oedematous properties, has the potential to reduce the degree of inflammation and oedema around the osteotomy site, which are important factors in the pathogenesis of neurosensory disorders. The topical application of dexamethasone may allow for a more concentrated therapeutic effect without the systemic side effects typically observed with its oral or parenteral administration. In addition, the lack of sufficiently developed clinical guidelines for the prevention and treatment of postoperative neurosensory disorders in BSSO indicates the need for further research in this area. Studying the efficacy of topical dexamethasone may help determine optimal surgical and postoperative treatment protocols, which will ultimately reduce the incidence of complications and improve treatment outcomes. The results of this study demonstrate a significant difference in the rate of recovery of inferior alveolar nerve IAN function between the experimental and control groups after sagittal split mandibular osteotomy BSSO. In the experimental group, where dexamethasone was applied topically, sensation recovery began as early as 1 week after surgery, and full recovery occurred by 3 months. In the control group, where dexamethasone was used only in systemic treatment, full sensation recovery was observed only by 6 months.

The practical significance of this is that topical application of dexamethasone can significantly shorten the period of neurosensory recovery after BSSO. This is especially important for patients, as rapid recovery of sensation improves postoperative quality of life, reduces discomfort, and speeds up rehabilitation. Restoration of inferior alveolar nerve function in a shorter period of time reduces the risk of long-term or permanent neurosensory impairment, which is a significant advantage for surgical practice. However, further studies with larger patient samples and longer follow-up periods are needed to fully confirm these results. This will help not only to more accurately assess the effectiveness of dexamethasone, but also to clarify the mechanisms of its action on the restoration of nerve function after jaw surgery.

The study analysed the properties of dexamethasone and the basic principles of pharmacokinetics. Dexamethasone is rapidly absorbed into the bloodstream after oral or injected administration. It is widely distributed in the body, penetrating into various tissues, including the brain and placenta. It is metabolised in the liver by cytochrome P450 enzymes. It is excreted mainly through the kidneys in the form of metabolites and lasts about 3–4 h in plasma, but the duration of action at the cellular level is much longer. This topic was also studied by J. Wen et al. [31]. The researchers analysed the pharmacokinetics of dexamethasone in obese children and adolescents. They emphasised that the metabolism of the drug changes due to physiological differences associated with obesity, which can affect the efficacy and safety of therapy. The researchers emphasised the need for an individual approach to dosing dexamethasone for this group of patients, as standard doses may be insufficient or cause side effects, and the authors of the current study agree with this statement. The study also analysed the effect of dexamethasone on osteoclasts, and it was determined that recovery with topical application occurred within one week, and without the use of the drug up to 3 months. This topic was also investigated by F. Umrath et al. [32]. The researchers investigated the effect of dexamethasone on osteogenesis, extracellular matrix, and secretion of osteoclastogenic factors of mesenchymal stem cells derived from the jaw periosteum. The researchers have concluded that dexamethasone stimulates the differentiation of these cells into bone cells, but may also promote the secretion of factors that activate osteoclasts that destroy bone, a statement that can be agreed upon. This also points to possible difficulties in balancing the stimulation of bone formation with the possible risk of bone resorption under the influence of dexamethasone.

Dexamethasone has been shown to cause immunological reactions in the body. Dexamethasone reduces the activity of the immune system, which can reduce inflammation, but also increases the risk of infections. Glucocorticosteroids, including dexamethasone, suppress the production of pro-inflammatory cytokines (e.g. interleukins) and increase the production of anti-inflammatory cytokines. The drug can lead to a decrease in the number of T-lymphocytes, especially CD4+, which reduces the immune response. In some cases, allergic reactions to dexamethasone are possible, although this is rare. They can manifest as skin rashes or anaphylactic reactions. It can also affect metabolism, which, in turn, can affect the immune response (e.g. by leading to hyperglycaemia).These reactions can vary depending on the dose, duration of treatment and sensitivity of the individual patient. This topic has also been studied by S.M.M. Al Chalabi et al. [33]. The researchers investigated the biochemical and immunological responses to dexamethasone in rats. The results showed that dexamethasone affects metabolism, causing changes in the levels of certain biomarkers and modulating the immune response, which is a statement that can be agreed upon. This study helps to better understand the mechanisms of action of this drug, especially its anti-inflammatory properties and potential side effects with long-term use.

The study analysed the effectiveness of dexamethasone and proved its importance in the preoperative and postoperative period. This issue was also considered by U. Fernández-Martín et al. [34], L. Posolenyk [35]. The researchers investigated the effect of preoperative administration of dexamethasone compared to methylprednisolone in patients undergoing lower third molars extraction. In a randomised control trial, the reduction of pain, swelling, and the need for painkillers after surgery were investigated. The results showed that both drugs are effective, but dexamethasone may provide better results in reducing inflammation and pain, a statement that can be agreed upon. The study highlights the importance of drug choice in improving postoperative care.

The study analysed the osteotomy technique and its features. This is a surgical procedure that involves cutting and shaping the bone. There are different types, for example, sagittal and transverse, which determines the technique. Surgeons must consider the proximity of important structures (nerves, blood vessels) to avoid complications. Preliminary diagnostics, including images (CT scans, panoramic radiographs) are required. General or local anaesthesia is used depending on the extent of the operation. After osteotomy, it is important to properly fix the bone fragments to ensure their fusion [36, 37]. It is also important to remember that postoperative care and physiotherapy are critical to restoring function. This issue was also studied by A.D. Al-Dawoody et al. [38], A. Tymchenko [39]. The researchers investigated the effect of mandibular mandibular osteotomy on the pattern of lingual split osteotomy during sagittal frame split osteotomy. The researchers compared the results and complications of different osteotomy techniques. The study showed that lower margin osteotomy can alter the split pattern, which, in turn, affects stability and healing. This emphasises the importance of technique selection to achieve optimal results in jaw surgery.

Topical application of dexamethasone in sagittal split osteotomy of the mandible shows significant results in minimising postoperative neurosensory disorders. Dexamethasone, due to its anti-inflammatory properties, can reduce swelling and inflammation, which helps to reduce pressure on the nerves. This has a positive effect on the speed of sensation recovery and reduces the risk of long-term complications. However, further studies with larger samples and longer monitoring are needed to confirm these findings. Given the effectiveness of dexamethasone in clinical practice can improve the outcomes of patients undergoing this procedure.

Limitations of the study included a small sample size and a relatively short follow-up period. A larger sample and longer follow-up period may provide even more data for the development of new therapeutic approaches. This study also opens up prospects for the use of dexamethasone in other surgical interventions involving nervous structures, thereby improving postoperative treatment in various fields of medicine. Thus, the introduction of such methods not only improves clinical outcomes, but also makes a significant contribution to the development of modern medicine and surgical practice, making the treatment process more effective and safe for patients.

## Conclusions

To sum up, it is possible to establish that the function of the IAN in experimental group began to restore from 1 week and normal sensitivity was fully achieved by third month, while in the control group sensitivity recovered completely up to sixth month. This is evidenced by the p values obtained for specified sensory test terms, indicating the level of statistical significance of the differences between the two groups. Thus, the findings confirm the effectiveness of proposed method of topical application with Dexamethasone and opens up prospects for the updated function of the IAN following the BSSO. Further research with larger sample sizes and longer follow-up periods is warranted to validate the findings of this study and elucidate the mechanisms underlying the beneficial effects of Dexamethasone on sensory recovery in patients undergoing BSSO.

It can be concluded that topical application of dexamethasone significantly accelerates the recovery of sensation of the lower alveolar nerve after osteotomy. This is important for patients because it shortens the period of recovery of nerve function, reducing the duration of postoperative complications such as numbness or discomfort. The relevance of this study lies in the fact that it points to the possibility of optimising surgical treatment through more effective management of tissue repair. In addition, the results highlight the potential for further research into the mechanisms of dexamethasone’s action on nerve regeneration.

## Data Availability

No datasets were generated or analysed during the current study.

## Abbreviations

IAN: Inferior alveolar nerve
BSSO: Bilateral sagittal split osteotomy
NSD: Neurosensory disorders
LT: Light touch test
St: Stomion
Ms: Mentolabial sulcus
Gn: Gnation
Ch: Chelion
M ± SD: Mean value and the standard deviation
Me: Median values
